# A systematic and prospectively validated approach for identifying synergistic drug combinations against malaria

**DOI:** 10.1186/s12936-018-2294-5

**Published:** 2018-04-11

**Authors:** Yasaman KalantarMotamedi, Richard T. Eastman, Rajarshi Guha, Andreas Bender

**Affiliations:** 1 Centre for Molecular Informatics, Department of Chemistry, University of Cambridge, Lensfield Road, Cambridge, CB2 1EW UK; 2 Division of Preclinical Innovation, National Center for Advancing Translational Sciences, National Institutes of Health, 9800 Medical Center Drive, Rockville, MD 20852 USA

**Keywords:** Synergy prediction, Malaria, Machine learning, Compound combination modelling, Transcriptional drug repositioning, Synergistic anti-malaria compound combinations

## Abstract

**Background:**

Nearly half of the world’s population (3.2 billion people) were at risk of malaria in 2015, and resistance to current therapies is a major concern. While the standard of care includes drug combinations, there is a pressing need to identify new combinations that can bypass current resistance mechanisms. In the work presented here, a combined transcriptional drug repositioning/discovery and machine learning approach is proposed.

**Methods:**

The integrated approach utilizes gene expression data from patient-derived samples, in combination with large-scale anti-malarial combination screening data, to predict synergistic compound combinations for three *Plasmodium falciparum* strains (3D7, DD2 and HB3). Both single compounds and combinations predicted to be active were prospectively tested in experiment.

**Results:**

One of the predicted single agents, apicidin, was active with the AC50 values of 74.9, 84.1 and 74.9 nM in 3D7, DD2 and HB3 *P. falciparum* strains while its maximal safe plasma concentration in human is 547.6 ± 136.6 nM. Apicidin at the safe dose of 500 nM kills on average 97% of the parasite. The synergy prediction algorithm exhibited overall precision and recall of 83.5 and 65.1% for mild-to-strong, 48.8 and 75.5% for moderate-to-strong and 12.0 and 62.7% for strong synergies. Some of the prospectively predicted combinations, such as tacrolimus-hydroxyzine and raloxifene-thioridazine, exhibited significant synergy across the three *P. falciparum* strains included in the study.

**Conclusions:**

Systematic approaches can play an important role in accelerating discovering novel combinational therapies for malaria as it enables selecting novel synergistic compound pairs in a more informed and cost-effective manner.

**Electronic supplementary material:**

The online version of this article (10.1186/s12936-018-2294-5) contains supplementary material, which is available to authorized users.

## Background

While recently progress has been made towards the reduction of malaria related morbidity and mortality, an increasing at-risk population and insecticide resistant vectors hamper eradication efforts. In 2015, nearly half of the world’s population (3.2 billion people) were at risk of contracting malaria, and 97 countries and territories had ongoing malaria transmission [1]. Most malaria cases and deaths occur in sub-Saharan Africa [1]; however, Asia, Latin America, and, to a lesser extent, the Middle East and parts of Europe are also at risk. In particular, the emergence and spread of drug-resistant parasites continues to limit the effective lifespan of current anti-malarial drugs [2].

Drug resistance has reduced the effectiveness of previous standard therapies for malaria, such as chloroquine and sulfadoxine–pyrimethamine [3, 4]. Today, artemisinin-based combination therapy (ACT) is frequently utilized, nearly universally in endemic regions, to reduce the selection of drug-resistant parasites [2]. Currently, five artemisinin-based combinations (ACT) are available for the treatment of uncomplicated *Plasmodium falciparum* malaria, namely artemether and lumefantrine, artesunate and amodiaquine, artesunate and mefloquine, dihydroartemisinin and piperaquine, as well as artesunate and sulfadoxine–pyrimethamine [5]. New combinations (such as artesunate and pyronaridine) have also recently been registered for use in particular countries [5]. In order to avoid drug resistance, combination therapies comprised of single agents with different modes of actions are usually indicated. However, this is not always true since among the ACT medicines, some combinations such as mefloquine and lumefantrine act on similar pathways to those of artemisinin-derived drugs [6]. Recently, reduced response to ACT has been observed which indicates an urgent need for new combination therapies [7].

**Fig. 1:**
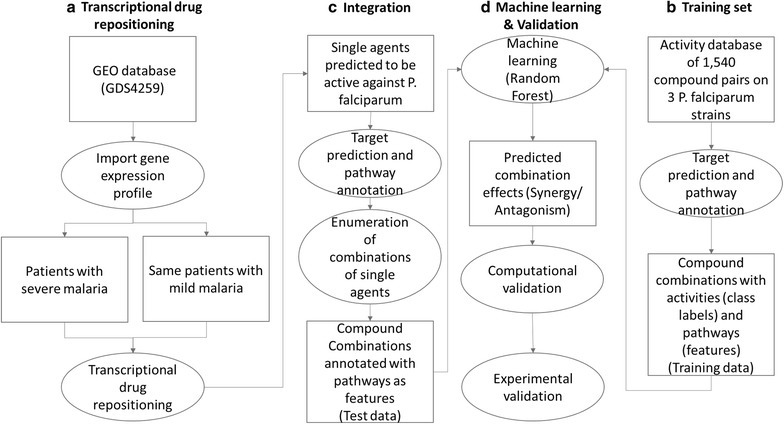
Data analysis work flow for predicting active compounds against malaria. The computational approach benefits from both transcriptional drug repositioning and machine learning. **a** The transcriptional drug repositioning approach imports gene expression data from GEO dataset (GDS4259) and compares patients with severe malaria *vs* patients with mild malaria to get gene expression profile of malaria. Transcriptional drug repositioning approach developed in this work is then used to predict potentially active single agents. **b** The machine learning part is trained on a dataset of activity of 1540 compound combinations applied on three different malaria *P. falciparum* strains. Target prediction and pathway annotation is used to define the features. **c** All combinations of potentially active single agents were annotated with targets and pathways and used as a test set input to the machine learning model built. **d** Activity of all possible combinations were predicted and computationally validated. All possible combinations were also experimentally validated and full accuracy of the algorithm in practice was evaluated

In this regard, a high-throughput combination screening study has recently been performed for malaria to provide experimental evidence for the efficacy of combinations [6]. However, due to the combinatorial explosion of the number of combinations possible, using a computational rationale for optimal selection of compound combinations can save cost and effort by providing a way to prioritize subsets of combinations from many possible compound pairs. Such compound combination modelling approaches have recently emerged for other diseases, particularly cancer [8, 9]; however there have been relatively few studies focusing on malaria. The studies which have been published include in silico screening of targets and molecular docking to find novel anti-malarial agents [10–12], and a drug synergy visualisation tool for malaria [13]. The machine learning approaches applied in the malaria field are usually for diagnostic purposes [14], or have other aims such as improving docking scores [15] and identifying a malaria transmission model [16]. However, this excludes learning from large scale in vitro malaria screening data to predict novel anti-malarial combination therapies, which is the aim of this study. In this work, a novel systematic approach is proposed for identifying compounds that boost human response to severe malaria and combination of compounds that show synergy in the malaria in vitro system (Fig. 1). The former was achieved via a transcriptional drug repositioning approach for shortlisting single agents and the later was achieved with a machine learning approach trained on in-vitro malaria screening data.

Transcriptomic data has been used previously for drug repositioning [17, 18], as well as for the selection of combination therapies in other areas [19]. There have been some transcriptional drug repositioning studies published for infectious diseases; [20, 21] however it has not been previously employed for malaria to the best knowledge of the authors. The usage of transcriptional data for compound selection is generally based on the hypothesis that compounds that affect differentially expressed genes of a disease in the opposite way to the disease itself (‘anti-correlated gene signatures’) have therapeutic potential for treating that particular disease. Experimental evidence supporting this approach has been presented in several studies, such as the repositioning of the antiulcer drug cimetidine for lung cancer [22], and of the antiepileptic topiramate for inflammatory bowel disease (IBS) [22]. In the present work, the human response to severe malaria has been derived in a different way compared to previous studies, namely gene expression data of blood samples of children with severe malaria was compared to gene expression signatures of the same children presented 1 month later, with mild malaria symptoms. Next, 201,776 compound gene signatures extracted from the recently published LINCS database [23] were compared to the malaria severity signature. This leads to a rank ordered list of compounds with anti-correlated gene signatures with malaria severity signature.

In this work, apart from the novelty of the application of transcriptional drug repositioning to malaria, previous approaches are also extended by utilising pathway annotations and in silico target predictions [24, 25] to the compound selection step. In this way, both biological readouts and on target bioactivity information are used to increase the signal and to have a more accessible understanding of compound action at hand. While understanding gene expression signatures can be rather complex, involving the up- and downregulation of pathways as well as the activity on individual proteins can often be more readily understood.

To this end, the in vitro combination screening data from 1540 compound pairs against three *P. falciparum* laboratory strains (3D7, DD, HB3) were utilized as a training set for the machine learning approach. To integrate the transcriptional drug repositioning, the machine learning and pathway annotation approach, synergistic combination pairs were selected from the single agents that were discovered in the preceding transcriptional drug repositioning approach. The activity predictions of individual compounds, and subsequently compound pairs, have been followed by prospective experimental validation, which resulted in the identification of novel single agent and synergistic compound combinations against *P. falciparum*. Hence, the results suggest that this method may be useful for prioritising new drug combinations for treating malaria.

## Methods

### Data

Training data previously generated at NCATS [6] included 1540 combinations of 56 individual compounds measured on three different *P. falciparum* strains, namely 3D7, DD2 and HB3 (https://tripod.nih.gov/matrix-client/?p=183, with assay IDs: 1463, 1464, 1465). For each of the combinations, the γ measure was calculated to characterize synergy between the pairs as described previously [26]. Values for *γ* < 1 represent synergy, *γ* = 1 represents additivity and *γ* > 1 represents antagonism. Combination screening quality is characterized by a per-combination QC metric, whose value ranges from 1 to 18, where 1 represents the highest quality data and 18 represents lowest data quality. Only data points with QC values less than 3 were used for subsequent analyses.

### Transcriptional drug repositioning approach

In order to select new compounds for screening, first a combined bioinformatics and cheminformatics approach developed earlier [27] was used to predict single compounds with activity against malaria. For this purpose GEO dataset [28] GDS4259 was downloaded using the GEOImporter [29] tool of GenePattern [30]. This dataset contains data derived from peripheral blood samples from five Malawian children with severe *P. falciparum* malaria, compared to the same children who presented with a mild case of malaria 1 month later [31]. This comparison gives rise to the gene signature of severe malaria, and it was used in this work as a disease signature. The controls were chosen as human host cells that have responded in a curative manner to the disease, and hence decreased disease severity. This choice was made to make sure that the host response is present in the malaria severity signature. In the next steps, it was searched for drugs to reverse this severity signature which is expected to change a state of severe malaria to convalescent and help boost the host response.

In order to find compounds that have strong negative connectivity (anti-correlation) with the gene signature of malaria severity, compound-gene expression profiles from the LINCS database (Phase I, GEO dataset GSE92742) [23] were utilized. This calculation was performed based on a modified Gene Set Enrichment Analysis (GSEA) [32]. Considering the top 50 and bottom 50 up- and down-regulated genes of every compound, the calculation derives a score describing the strength of connection of compound signatures with a given disease profile. The scoring system leads to a rank ordered list of compounds that are assumed to be able to reverse the gene expression signature present in the malaria severity signature. Fifty highly scored compounds were shortlisted as single agent candidates.

### Machine learning approach

The machine learning part of the algorithm involved in-silico target prediction, pathway annotation, synergy model generation and computational validation as follows.

### Target prediction

Firstly, protein targets of all the 56 compounds in the training dataset were predicted based on an updated version of target prediction algorithm developed previously [24]. The algorithm was retrained on compound/target pairs from ChEMBL [33] v17 and compound targets were predicted using a Laplacian Modified Naïve Bayes scoring system [25, 32]. Next, protein targets were predicted for the 50 shortlisted compounds from the transcriptional drug repositioning approach. The predicted protein targets with a score over 14 were selected as potential protein targets of each compound. The score cut-off gave rise on average to six targets for a compound, which is a reasonable number of targets for a ligand, based on previous analyses [34].

### Pathway annotation

Next, pathways enriched by the targets of each compound were identified. For this purpose, 2010 human pathways and their associated genes from the Biosystems [35] database were extracted. The equivalent Entrez gene IDs of each protein target of compounds were retrieved using Biomart [36]. For each compound a list of, on average, six gene IDs that the compound is predicted to interact with (the ‘compound gene signature’) was obtained. Then, the number of shared genes between each compound gene signature and gene members of each of 2010 pathways were found and stored as a raw integer score in a sparse vector, resulting in a vector with 2010 values for each compound, termed the ‘pathway signature of a compound’.

Next, for each combination of compound ‘a’ and compound ‘b’ in the combination dataset, a descriptor vector was created by merging their compound pathway signatures using the following equation:

1 $$ p_{i,a,b} = \left( {P_{i,a} + 1} \right)*\left( {P_{i,b} + 1} \right) $$

where *P*_*i*,*a*_ and *P*_*i*,*b*_ are the *i*th value in compound ‘a’ or ‘b’ pathway signature which is the number of shared genes between *i*th pathway and compound ‘a’ or ‘b’ gene signature. *p*_*i*,*a*,*b*_ is the *i*th value in the pathway signature of combination of compound ‘a’ and compound ‘b’. Each term was added with one to avoid *p*_*i*,*a*,*b*_ = 0 in cases where one of *P*_*i*,*a*_ or *P*_*i*,*b*_ equals zero. In this way, it is avoided to filter out the effect of one compound on the pathway when the other one has no effect on that pathway. In other words, the above formula gives a high score to pathways that are shared between both compounds which is the product of the score of both compounds, while also not neglecting the pathways that are only hit by one compound.

### Synergy model generation

Using the above formula, a training file was created for each pair of compound pathway signatures labelled with synergy or no synergy, depending on gamma and QC. Pairs of compounds with gamma value < 0.975 and QC ≤ 3 were marked as synergistic and gamma > 1.025 and QC ≤ 3 were marked as antagonistic. The 0.975 and 1.025 cut-offs for gamma were chosen to avoid the additivity window. All the data with QC > 3 was discarded. Next, the training file was used as input for a Random Forest [37] algorithm consisting of 200 trees as implemented in Matlab (2015B) using the TreeBagger [38] function. This algorithm was hence able to predict the synergy of a compound combination, based on the pathways that are hit by this given pair of compounds.

### Computational validation for drug combination

As a computational step to validate the approach prior experiments, cross validation was used to evaluate performance of the methods. The number of true positives (TP), false positives (FP) and false negatives (FN) were used to calculate precision ($\frac{\text{TP}}{{{\text{TP}} + {\text{FP}}}}$) and recall ($\frac{\text{TP}}{{{\text{TP}} + {\text{FN}}}}$). The tenfold cross validation was applied on the original data which was yielding 66% precision, 61% recall and 61% for the F-measure over the two classes of synergy and antagonism, averaged over all three strains.

### Prospective utilisation of the synergy model

Next, combinations of compounds in the shortlist produced using the above transcriptional drug repositioning approach were annotated with pathways using Eq. 1 and fed to the random forest model as a prospective test set (in order to predict likely synergistic compound combinations for this new set of compounds). The random forest model hence predicted synergies and antagonism for compound combinations, and predicted pairs were ranked by their probability of being synergistic. The top 17 compound combinations, representing 14 unique single agents, were selected for experimental validation as summarized in Additional file 1: Table S2.

### Experimental validation of compounds for malaria

The *P. falciparum* parasite lines were maintained in in vitro culture conditions as described previously [39]. Methods for the SYBR qHTS and calculation of IC_50_ and definition of curve classes have been used as in previous work [39–41], as was the plating of compounds for combination screening using acoustic dispense methods [42]. As shown in the Additional file 2: Figure S1, classes of activity were assigned based on growth inhibition curves where − 1.1 shows a complete curve as well as high efficacy, − 1.2 represents a complete curve but only partial efficacy, − 2.1 symbolizes a partial curve which however exhibits high efficacy, − 2.2 represents a partial curve with only partial efficacy and − 2.3 represents a partial curve with high efficacy, but only poor curve fit. All HTS assays were read at 72 h. Percent response values shown in matrix heat maps represent relative growth as obtained from SYBR Green fluorescence intensity values normalized to controls. Synergy metrics for combinations were computed as described [42]. Data generated as part of the prospective validation can be accessed at https://tripod.nih.gov/matrix-client/?p=1261.

## Results

### Prospective validation of single agents

**Fig. 2:**
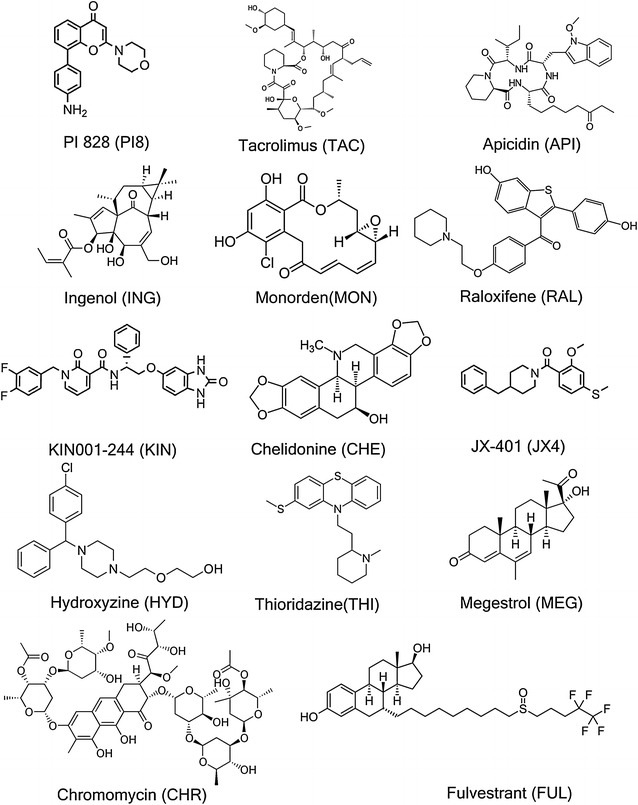
Chemical structures of predicted single agents against malaria. All the structures were experimentally tested in the *P. falciparum* screen (see Table 1 for results)

With the aid of the transcriptional drug repositioning approach, 14 single agents (structures are displayed in Fig. 2) were prioritized and subsequently tested experimentally for their activity against the 3D7, DD2 and HB3 strains of *P. falciparum*. The results of the screen are summarised in Table 1, where chromomycin-a3 (CHR), fulvestrant (FUL), apicidin (API), ingenol (ING) and tacrolimus (TAC) exhibited full inhibition in the dose response curves in low concentrations. Of those, chromomycin-a3, fulvestrant and apicidin were active in nanomolar doses in all the three strains tested, with the average AC50 values of 11, 67 and 78 nM. JX-401 (JX4), raloxifene (RAL), hydroxyzine (HYD), thioridazine (THI), KIN001-244 (KIN), PI 828 (PI8), megestrol (MEG) exhibited complete or partial activity in different individual strains. Monorden (MON) and chelidonine (CHE) exhibited partial or single dose activity across all strains and were not progressed further. All experimental results data can be accessed at https://tripod.nih.gov/matrix-client/?p=1261.

**Table 1:** Experimental validation of predicted anti-malarial single agent compounds

Compound	Class 3D7	Class DD2	Class HB3	AC50 3D7	AC50 DD2	AC50 HB3	Average AC50
CHR	*−* *1.1*	*−* *1.1*	*−* *1.1*	*0.0119*	*0.0106*	*0.0106*	*0.011*
FUL	*−* *1.1*	*−* *1.1*	*−* *1.1*	*0.0335*	*0.0335*	*0.1332*	*0.0667*
API	*−* *1.1*	*−* *1.1*	*−* *1.1*	*0.0749*	*0.0841*	*0.0749*	*0.078*
ING	*−* *1.1*	*−* *1.1*	*−* *1.1*	1.3324	1.495	1.495	1.4408
MON	− 2.1	− 2.1	− 2.3	1.4956	1.0588	1.8828	1.4791
RAL	*−* *1.1*	*−* *1.1*	− 2.1	*0.7493*	*0.3347*	4.7277	1.9372
TAC	*−* *1.1*	*−* *1.1*	*−* *1.1*	1.8821	3.7553	1.6774	2.4383
CHE	1.3	− 2.3	1.3	*0.0266*	10.5839	*0.0011*	3.5372
JX4	*−* *1.1*	*−* *1.1*	− 2.1	0.7493	*0.0595*	10.5839	3.7976
HYD	*−* *1.1*	*−* *1.1*	− 2.1	2.6586	1.6774	11.8754	5.4038
THI	− 2.1	− 2.1	− 2.1	10.5839	14.9502	14.9502	13.4948
MEG	− 2.1	− 1.1	− 2.1	14.9502	1.8821	23.6945	13.5089
PI8	− 2.3	− 2.1	− 2.1	13.3244	11.8754	16.7744	13.9914
KIN	− 2.1	− 2.1	− 2.1	13.3244	21.1177	16.7744	17.0722

The fitted AC50 values (µM) for the single drugs as well as classes of activity are listed. AC50 indicates 50% maximal response (for inhibition or agonism) and is calculated by fitting a 4-parameter logistic model to the concentration, response data. Curve classes are defined in Additional file 2: Figure S1

Classes that are identified as −1.1 are highlighted in italics as it signifies complete curve as well as high efficacy

### Prospective validation of drug combinations

Thirty five compound pairs were selected based on synergy prediction model as well as single agent screening data and were experimentally tested against *P. falciparum* strains of 3D7, DD2 and Hb3. Table 2 represents predicted probability for synergy of each of combinations in each strain (calculated before experiments) as well as synergy metric, γ [26] values, (calculated after experiments). Among 35 combination pairs that have been tested in this study, 28 were predicted to be synergistic at least in one strain. Among those 28 predicted pairs, 27 were showing mild-strong synergy, 17 were showing moderate-strong synergy and 8 were showing strong synergy at least in one strain. As one can see in the Table 3, only one of five currently used ACT medicines (mefloquine–artesunate) shows strong synergy on all the three strains and one (amodiaquine–artesunate) has strong synergy only on the HB3 strain. Precisions and recall measures were utilized to measure the accuracy of synergy predictions compared to experiments, the results of which are shown in Table 4. For this purpose various gamma cut-offs were used to signify mild-to-strong (gamma ≤ 0.995), moderate-to-strong (gamma ≤ 0.975) and strong (gamma ≤ 0.95) synergies. The overall average precision and recall of experiments over the three strains were 83.5 and 65.1% for mild-to-strong, 48.8 and 75.5% for moderate-strong and 12.0 and 62.7% for strong synergies.

**Table 2:** Synergy prediction and synergy scores based on experiments

Compund 1	Compound 2	3D7 Pred	3D7 Gamma	DD2 Pred	DD2 Gamma	HB3 Pred	HB3 Gamma
TAC	HYD	0.309	0.948**	0.189	0.885**	*0.501*	0.951*
FUL	TAC	0.627	0.961*	*0.612*	0.908**	*0.659*	*0.989*
ING	TAC	*0.820*	0.954*	*0.674*	0.917**	*0.854*	0.954*
FUL	MEG	*0.722*	1.007	*0.583*	0.918**	*0.745*	0.970*
MEG	HYD	*0.784*	*0.985*	*0.629*	0.920**	*0.738*	*0.988*
KIN	HYD	0.475	*0.984*	0.371	0.938**	0.477	*0.977*
API	TAC	0.416	0.975*	*0.685*	0.942**	*0.523*	0.946**
FUL	RAL	*0.792*	*0.980*	*0.739*	0.952*	*0.825*	0.966*
API	HYD	*0.806*	*0.987*	*0.717*	0.952*	*0.501*	0.968*
FUL	THI	*0.651*	*0.982*	*0.714*	0.954*	*0.540*	0.960*
THI	RAL	*0.549*	0.931**	*0.698*	0.956*	0.375	0.911**
PI8	HYD	0.278	*0.977*	0.293	0.961*	0.171	*0.993*
FUL	HYD	*0.788*	0.965*	*0.747*	0.962*	*0.734*	0.951*
TAC	RAL	0.483	*0.995*	0.342	0.964*	*0.612*	0.962*
ING	JX4	*0.859*	*0.980*	*0.740*	0.968*	*0.826*	0.975*
MEG	RAL	*0.795*	0.937**	*0.648*	0.969*	*0.791*	0.966*
ING	RAL	*0.836*	0.964*	*0.658*	0.975*	*0.793*	0.966*
API	RAL	*0.814*	0.997	*0.697*	*0.976*	0.472	*0.992*
CHR	HYD	*0.633*	1.001	0.238	*0.979*	*0.644*	*0.991*
KIN	JX4	*0.510*	*0.987*	*0.530*	*0.982*	0.479	*0.989*
API	JX4	*0.761*	1.020	*0.723*	*0.984*	0.495	0.970*
CHR	TAC	0.418	*0.99*	0.163	*0.987*	0.445	0.996
JX4	HYD	0.498	*0.984*	*0.503*	*0.987*	0.498	*0.986*
PI8	ING	*0.551*	0.975*	0.443	*0.991*	0.499	*0.989*
PI8	JX4	0.238	*0.990*	*0.621*	*0.994*	0.214	*0.981*
PI8	RAL	0.369	1.014	0.293	0.997	0.126	*0.995*
CHR	JX4	*0.709*	*0.990*	0.355	0.999	*0.608*	*0.991*
API	MEG	*0.776*	*0.981*	*0.632*	1.004	*0.582*	*0.979*
CHR	MEG	*0.817*	*0.995*	*0.568*	1.010	*0.791*	*0.985*
PI8	TAC	0.283	*0.986*	0.205	1.014	0.118	*0.983*
API	CHR	*0.785*	0.999	*0.722*	1.014	*0.525*	0.998
CHR	RAL	*0.785*	*0.990*	0.442	1.019	*0.695*	1.004
PI8	MEG	0.491	0.972*	0.366	1.021	0.272	0.966*
ING	HYD	*0.869*	*0.985*	*0.699*	1.021	*0.810*	*0.992*
PI8	KIN	0.246	*0.989*	0.348	1.046	0.197	0.997

This table represents gamma (γ) values as a metric for calculation of synergy as well as predicted probability of synergy for three different *P. falciparum* strains. Values of γ ≤ 0.95 represent strong synergy and were marked with ** and italic. Values of 0.95 < http < 0.975 were identified as moderate synergy and marked with * and italic, 0.975 < γ < 0.995 were identified as mild synergy and marked with italic font. Prediction scores above 0.5 were true predictions and were marked with italic font

**Table 3:** Gamma synergy values for ACT as current synergistic therapies

Compound 1	Compound 2	3D7 Gamma	DD2 Gamma	HB3 Gamma
Mefloquine	Artesunate	0.911**	0.786**	0.797**
Amodiaquine	Artesunate	*0.993*	1.064	0.955*
Sulfadoxine	Artesunate	0.986	1.016	0.936**
Lumefantrine	Artemether	2.221	1.058	0.997
Piperaquine	Dihydroartemisinin (DHA)	0.999	1.007	0.999

This table shows synergy metric gamma for current anti-malarial combination therapies for comparison reason to the results provided in Table 2 (similar markings)

**Table 4:** Precision and recall calculated for synergistic compounds

Strain	Mild-to-strong synergy (gamma ≤ 0.995)				Moderate-to-strong synergy (gamma ≤ 0.975)				Strong synergy (gamma ≤ 0.95)			
	3D7	DD2	HB3	AVG	3D7	DD2	HB3	AVG	3D7	DD2	HB3	AVG
True positives	18	18	19	18.3	7	13	12	10.7	2	5	1	2.7
False negatives	11	7	12	10.0	3	4	3	3.3	1	2	1	1.3
False positives	5	4	2	3.7	16	9	9	11.3	21	17	20	19.3
Precision (%)	78.3	81.8	90.5	83.3	30.4	59.1	57.1	48.5	8.7	22.7	4.8	12.1
Recall (%)	62.1	72.0	61.3	64.7	70.0	76.5	80.0	76.2	66.7	71.4	50.0	66.7
F measure (%)	69.2	76.6	73.1	72.8	42.4	66.7	66.7	59.3	15.4	34.5	8.7	20.5
Training set synergies (%)	42.8	41.2	40.0	41.3	25.7	25.3	23.0	24.7	15.8	15.6	13.4	14.9

Precision and recall was calculated for each of the three *P. falciparum* strains with respect to three different cut-offs for gamma to distinguish the performance of the algorithm for mild-to-strong, moderate-to-strong and strong synergy. As you can see in all of the predictions recall has been kept high between 50 and 80% depending the expected synergy level and the strain. The highest precision is for Mild-to-strong synergies ranging from 61.3 to 72%. However, as the synergy level expectation is increased to Moderate-to-strong or only Strong synergies the Precision drops to 30–59% and 4.8–22.7%

**Fig. 3:**
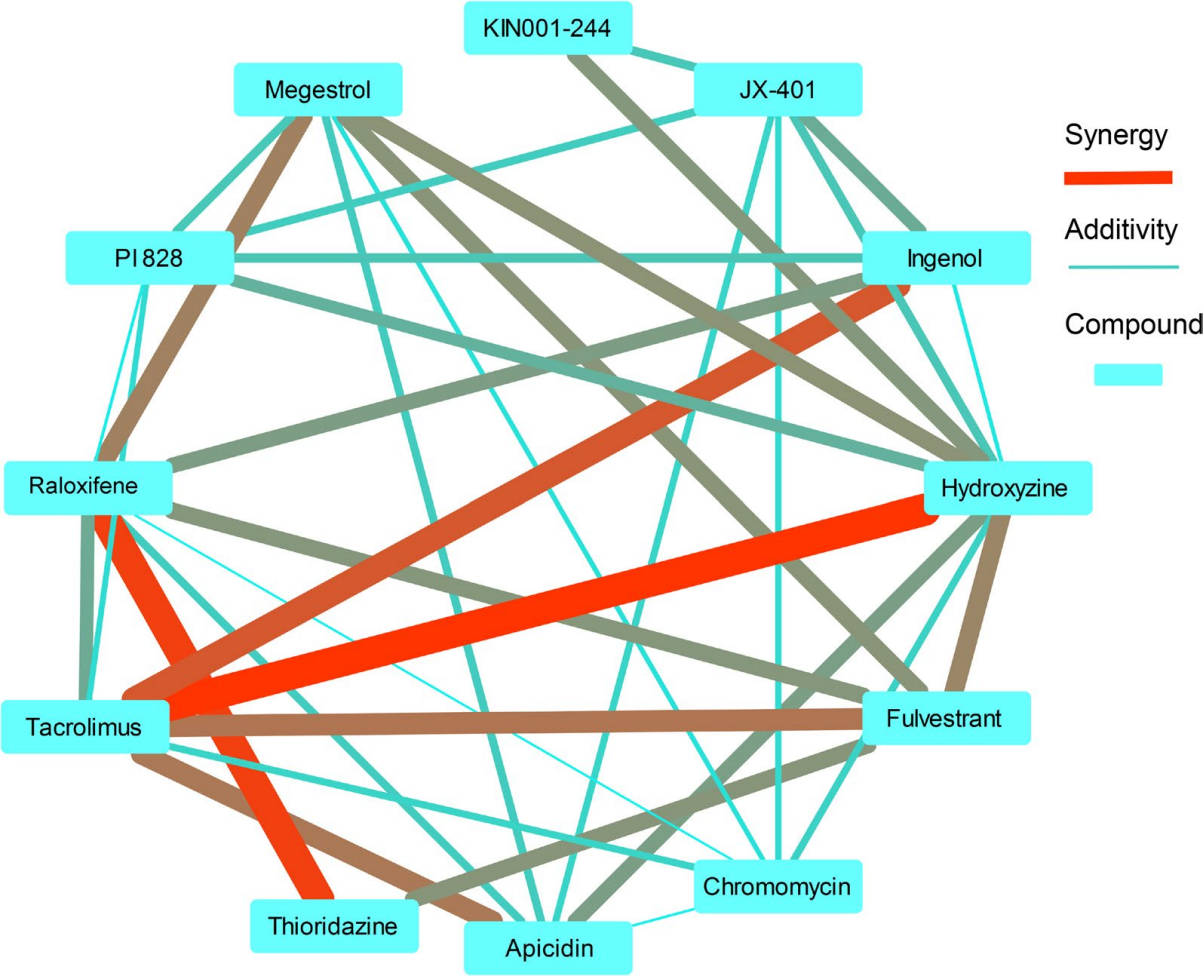
Network representation of compound combination synergies. Red thick edges represent high synergy and blue thin edges represent additivity. The level of thickness or redness is inversely related to gamma (calculated based on experiments) in average for the tree *P. falciparum* strains used in this study. Tacrolimus-hydroxyzine, raloxifene-thioridazine, represent highest synergistic compound pairs followed by ingenol-tacrolimus, tacrolimus-fulvestrant, tacrolimus-apicidin and raloxifene-megestrol pairs. Tacrolimus is synergistic with highest number of compounds (three)

A network representation of compound pairs were provided in Fig. 3 to represent synergy strength of different compound pairs and identify compounds that have highest number of synergies with other compounds. It can be seen that tacrolimus-hydroxyzine and raloxifene-thioridazine, represent the most synergistic compound pairs followed by ingenol-tacrolimus, tacrolimus-fulvestrant, tacrolimus-apicidin and raloxifene-megestrol pairs. Tacrolimus is synergistic with highest number of compounds (three).

**Fig. 4:**
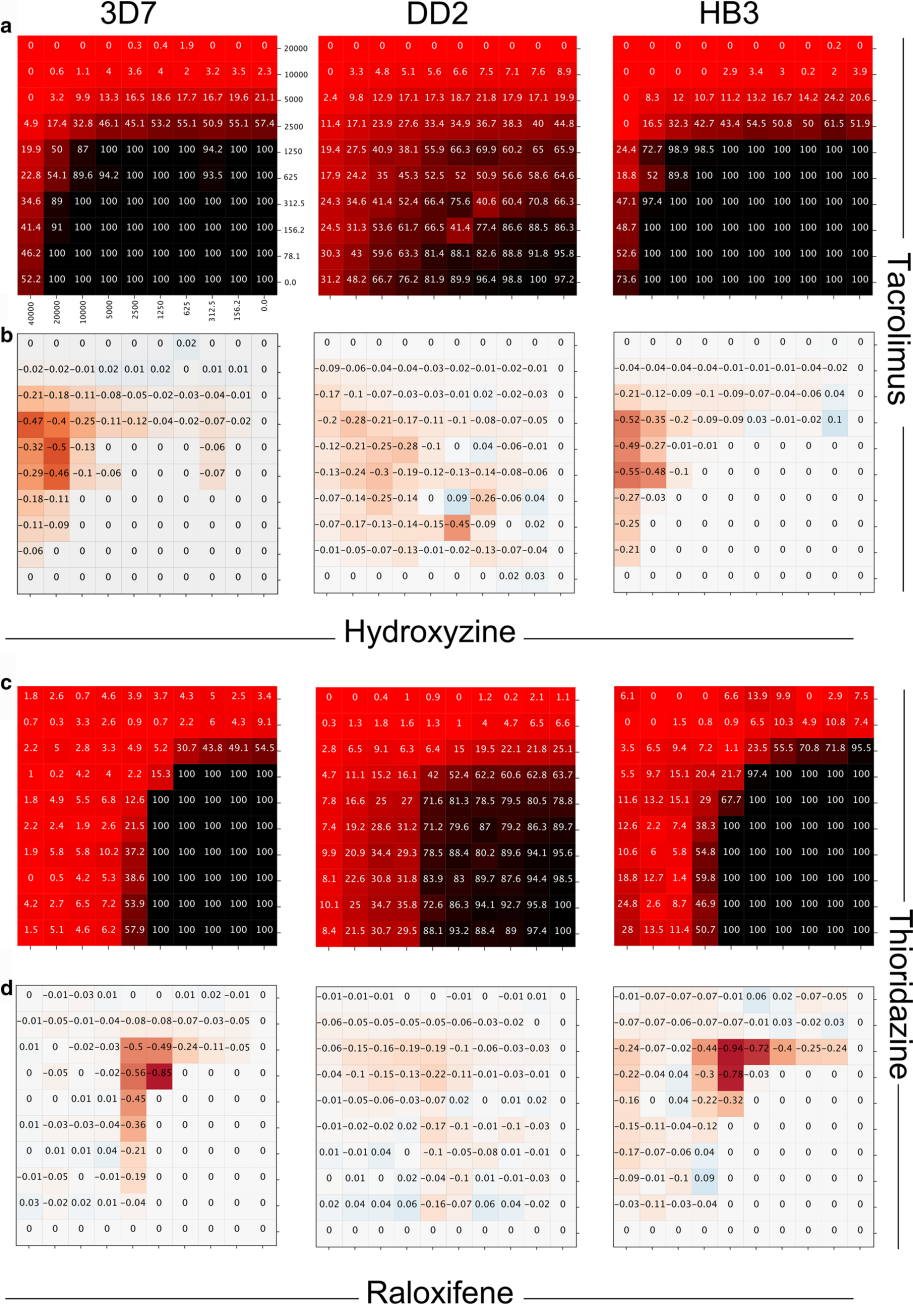
Synergistic compound combinations identified against *P. falciparum*. Representation of dose–response matrix and HSA synergy model for tacrolimus-hydroxyzine (**a**, **b**) and raloxifene-thioridazine (**c**, **d**) in the three parasite lines (3D7, DD2, HB3). The effect of increasing drug concentration from right to left and bottom to top was assessed in a 10 × 10 block size matrix (**a**, **c**). HSA synergy model represents in which concentrations maximum synergy occurs (**b**, **d**)

The experimental validation of the most synergistic compound combinations in the *P. falciparum* strains were depicted in Fig. 4 using the HSA synergy model [43] to identify the optimal synergy doses. In the DD2 assay, the most synergistic combination was tacrolimus with hydroxyzine, where 1.250 µM hydroxyzine and 0.156 µM tacrolimus demonstrated highest synergy of − 0.45 in the HSA system (Fig. 4b). In the 3D7 and HB3 strains on the other hand, raloxifene together with thioridazine was the most synergistic compound pair (Fig. 4d). Here for the 3D7 strain 1.250 µM raloxifene and 2.5 µM thioridazine achieved maximum synergy of − 0.85, while in the HB3 assay this is the case for concentrations of 2.5 µM of raloxifene and 10 µM of thioridazine with synergy of − 0.94. However, high synergy values were also observed at other compound concentrations, such as 2.5 µM of raloxifene with 5 µM of thioridazine, as well as 1.25 µM of raloxifene and 10 µM thioridazine with synergy values of − 0.78 and − 0.72, respectively.

## Discussion

### Mechanistic justification of the compounds

In this section, differentially expressed genes and pathways that are involved in the malaria severity signature as well as potential modes of action on the target and pathway level are discussed. It is important to consider that human targets and human pathways are used as the gene expression data utilized is also from human sources. This will enable studying the effect of the compounds on the host.

In the malaria severity signature, several immune response related genes were down-regulated. In other words, these genes were up-regulated in patients that responded to the disease in a curative way and hence recovered from the disease. The computational transcriptional drug repositioning approach is looking for compounds that reverse the malaria severity signature and up-regulate the immune response genes to help boost the immune response. The immunity related genes that are down-regulated in the malaria severity signature are: PRF1, GNLY, OAS2, MX1, OAS3 and CCL5. PRF1 encodes a protein with structural similarities to complement component C9 that is important in immunity [44]. GNLY protein is present in cytotoxic granules of cytotoxic T lymphocytes and natural killer cells, and it has antimicrobial activity against *M*. *tuberculosis* and other organisms [44]. OAS2 and OAS3 encode a members of the 2–5A synthetase family, essential proteins involved in the innate immune response to viral infection. These molecules activate latent RNase L, which results in viral RNA degradation and the inhibition of viral replication. They play significant role in the inhibition of cellular protein synthesis and viral infection resistance [46]. MX1 participates in the cellular antiviral response. CCL5 is one of several chemokine genes. Chemokines form a superfamily of secreted proteins involved in immunoregulatory and inflammatory processes. SLPI is also up-regulated in the malaria severity signature. Its inhibitory effect contributes to the immune response [44]. Pathways associated with mild malaria include the type I interferon-mediated signalling pathway, T cell activation and other pathways representing many aspects of immune activation [31]. The malaria severity signature contains six genes that were associated with severe malaria, including carbonic anhydrase 1 (CA1), G-protein-coupled receptor 89B (GPR89B), lipocalin 2 (LCN2), thymidine kinase 1 (TK1), small nucleolar RNA, C/D box 30 (SNORD30), and TBC1 domain family member 3 (TBC1D3) (P ≤ 0.05; fold change of ≥ 1.5) [31]. Thymidine kinase 1 was recently found to be a biomarker of cerebral malaria susceptibility in the murine model [45], and carbonic anhydrase, reflects the blood’s abnormal acid base environment during severe disease. Further discussion of genes and pathways in the malaria severity signature has been published before [31].

Among the most potent single agents, tacrolimus has targets with established links to malaria. Tacrolimus has been previously studied for its potential anti-malarial activity by binding to PfFKBP35 and PvFKBP35 proteins of the parasite [46, 47]; however, the synergistic combination with hydroxyzine has not been reported before. Tacrolimus as well as apicidin were predicted to target “TGF-beta receptor signalling” pathway of human host. It is known that tacrolimus enhances TGF-Beta expression [48] while production of this protein is inversely correlated with severity of murine malaria infection [49]. Activation of another member of this pathway namely latent TGF-beta is suggested as a novel mechanism for direct modulation of the host response by malaria parasites [50]. Hence, modulation of TGF-Beta signalling may be a novel mechanism of tacrolimus that directly affects the host. Moreover, in vivo validation of apicidin against *Plasmodium berghei* in mice is evident from the literature [51].

**Fig. 5:**
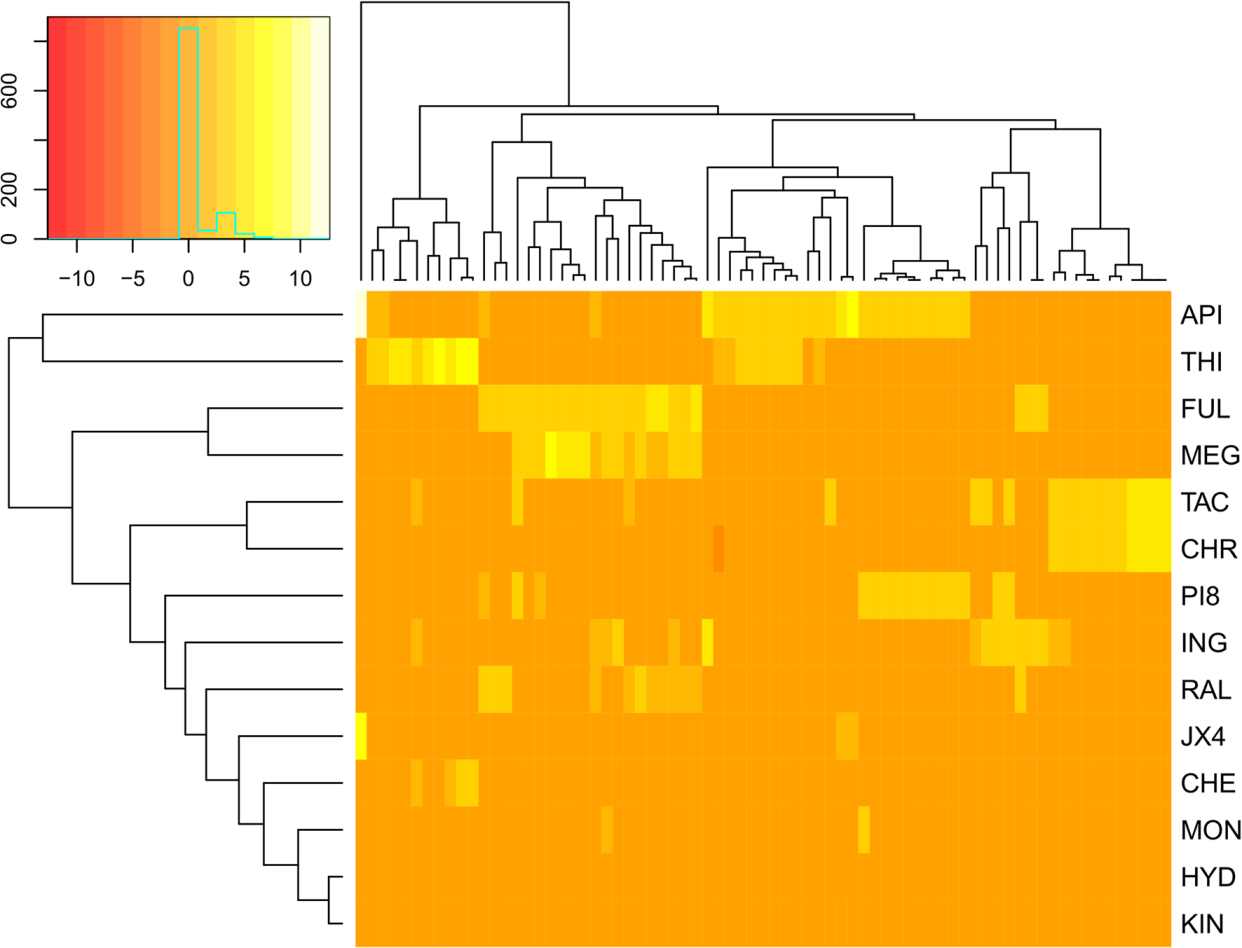
Pathway annotation of selected agents in the *P. falciparum* screen. Y axis pathway IDs are explained in Additional file 4: Table S1. Compounds are clustered based on their similarity in pathway bioactivity space, with yellow indicating high enrichment Z-score of a particular pathway, and red indicating lower enrichments

On the other hand, pathways enriched by the predicted targets of the selected single agents according to Biosystems pathways are depicted in Fig. 5 (also represented in Additional file 3: Figure S2 and listed in more detail in Additional file 4: Table S1). Figure 5 suggests that highest synergistic combinations such as tacrolimus with hydroxyzine (average (γ) = 0.928), as well as raloxifene and thioridazine (average (γ) = 0.932), target independent pathways rather than shared pathways. On the other hand, pairs of compounds that have the most similar pathway signature did not have strong synergy. As an example, tacrolimus-chromomycin, hydroxyzine-KIN001244 and fulvestrant-megestrol are pairs that are clustered together (Fig. 5) but all of them are showing week synergies with average γ values of 0.991, 0.965, and 0.966. The data suggests that targeting independent pathways by compound pairs is privileged to targeting the same pathway multiple times for synergy to emerge.

### Limitations of the study

The limitation of transcriptional drug repositioning approaches that are gaining popularity is that they cannot be applied for predicting compounds that act on parasite directly. Some recent other applications of transcriptional drug repositioning approaches for finding antivirals took into account the effect of compounds on host cells [20, 21]. This limitation is due to CMap and LINCS databases that are the gateways for transcriptional drug repositioning approaches. The LINCS database used in this work contains 201,776 compound treatment gene signatures that are applied on “human” cell lines and hence provides genes up/down-regulated in human tissue. Hence, it is not applicable to the parasites directly. Another limitation of the transcriptional drug repositioning approaches is that they are limited to the compounds that are included in LINCS or CMap database.

The use of transcriptional drug repositioning in the context of this project can facilitate identifying single agent compounds that help boost immunity of host to the parasite. Given that the combination of those compounds are expected to show synergy in the in vitro system as well, a two round selection process was performed. In the first round, single compounds were predicted to be active in blood cells of patients infected with severe malaria. In the second round, combinations of those single compounds were selected that were predicted to be synergistic on the parasite infected in vitro system containing host red blood cells. The final compound combinations were as a result of using both filtering methods. It needs to be considered that the in vitro system includes human red-blood cells and this means human pathways are present in the in vitro system. Moreover, the training data for synergies was generated based *on the same* in vitro platform of the final experimental tests which has human red blood cells infected with malaria parasite. Hence, the final combination sets are correctly predicted for the right platform.

One of the limitations of the study is the number of patient samples used in this work. Even though the gene expression samples of only five patients were used in the study, they were hand-picked among 119 patients participating in Blantyre malaria research. These patients were selected as they manifested clinical features of severe malaria but were dramatically recovered, having no clinical evidence of severe disease. They were also reported to be clear of peripheral blood parasites and bacterial meningitis at admission and after 48 h [31]. The authors that published the database claimed that “five severe/mild paired samples had sufficiently high-quality RNA for microarray and further analysis”. However, the sample size was mentioned as a limitation of the study.

Generally, the application of computational biology approach introduced in this study is limited to early stage drug discovery/repurposing of single agents as well as combination therapies. Any of the findings of such early stage studies should be followed up with further lab and possibly animal studies to be able to be translated into clinical trials. However, the approach can be used on other diseases as well given availability of appropriate data.

### Performance evaluation and effect of gamma choice

The choice of gamma affects sample size of the synergy class and hence it is a trade-off between the sample size and quality of samples (degree of synergy). Gamma cut-off of 0.975 marked in average between the three strains 379.3 out of 1540 combinations in the training set as synergistic while gamma cut-off of 0.95 reduced average number of synergistic instances to 229.7. Given the dimensionality of the data with 2010 features, higher gamma cut-off of 0.975 was chosen to keep the sample size in the synergy class as high as possible while avoiding the additivity window. To show that the gamma cut-off is relevant in this study, Table 3 shows gamma values of current combinational therapies. Only one of five currently used ACT medicines (mefloquine–artesunate) on all the three strains and one (amodiaquine–artesunate) on the HB3 strain have gamma values bellow 0.95. Hence, the cut-off chosen is appropriate, given the observed synergy even for established combination therapies.

To address the effect of gamma choice on the validation step, various gamma cut-offs were used and the accuracy of the algorithm based on each of the cut-offs were validated. Table 4 shows the precision and recall based on each of the cut-offs. When gamma cut-offs of 0.975 and 0.995 are used, the precision of the algorithm is 1.98 and 2.02 times better than the training set while recall has been kept as high as 75.5 and 65.1%. It is notable that the training data is selected in a biased way rather than randomly, as it includes current combination therapies and investigational drugs. In case of 0.95 cut-off, the average recall is kept still as high as 62.7% in average. The precision on the DD2 strain is 1.45 times better than the biased training set. However, in average over the three strains the precision is similar to the training set which was selected with biological insights. Hence the algorithm is almost twice better than biased selection for predicting mild-to-strong or moderate-to-strong synergies. In case of only strong synergy detection the approach is 1.4 times better than biased in the DD2 strain but similar to biased in average for the three strains. To the best of authors’ knowledge there is no similar study published elsewhere that has a better precision and recall for predicting synergistic pairs for malaria.

## Conclusions

Recently drug resistance has emerged, and continues to develop, to the existing drug combination therapies for malaria, and hence discovering novel therapies is of much importance [7]. However, testing combinations is costly and time consuming. Systematic approaches towards novel compound combination discoveries can reduce financial cost as it helps to identify compounds in a more informed manner. In this work, an integrated transcriptional drug repositioning and machine learning approach was developed to predict synergistic compound combinations for malaria. The transcriptional drug repositioning approach led to the identification of active single agents against malaria. Chromomycin-a3 (AC50 = 0.011 µM), fulvestrant (0.0667 µM), apicidin (0.078 µM), ingenol (1.4408 µM) and tacrolimus (2.4383 µM) exhibited full inhibition in low concentrations. The machine learning approach utilized here was trained on a dataset of experimentally assayed compound combinations (including synergistic, antagonistic and additive responses), across three strains of malaria to suggest new compound combinations.

The predicted synergistic compound combinations were experimentally validated, and synergistic compound combinations were identified with overall precision and recall of 83.5 and 65.1% for mild-to-strong, 48.8 and 75.5% for moderate-strong and 12.0 and 62.7% for strong synergies. In all of the predictions recall has been kept high between 50 and 80% depending the expected synergy level and the strain. Highest precision is achieved for mild-to-strong synergies ranging from 61.3 to 72%. However, as the synergy level expectation is increased to moderate-to-strong or only strong synergies the precision drops to 30–59% and 4.8–22.7%. Strong novel synergistic compound combinations including combination of tacrolimus with hydroxyzine (average (γ) = 0.928) as well as raloxifene and thioridazine (average (γ) = 0.932) were identified. Moreover, according to the data, targeting independent pathways was more privileged that targeting the same pathway with both compounds in a combination. For retrospective validation, it is notable that tacrolimus and apicidin that were predicted for *P. falciparum* strains have previously been studied for malaria. In vivo validation of apicidin against *P. berghe*i malaria in mice is evident from the literature [51]. This retrospective validation provides further assurance of the computational approach presented in this study.

For a single agent to be translated to actual cure, there are several requirements. One of these is that the dosage that activity is occurring, should be lower than maximal safe plasma concentrations of the drugs. The maximal plasma concentration of the single agents are provided in Table 5. In this study, the highest active single agents were apicidin, fulvestrant and chromomycin-a3. The only single agent that is active in lower doses than the maximal safe plasma concentration is apicidin. The AC50 value of apicidin is 74.9, 84.1 and 74.9 nM in 3D7, DD2 and HB3 *P. falciparum* strains while its maximal safe plasma concentration in human is 547.6 ± 136.6 nM. Apicidin at the dose of 500 nM kills in average 97% of the parasite while it is a safe dose for human. However, apicidin is not approved drug and is not in clinical trials. Hence, entering clinic requires further safety and efficacy studies of apicidin.

**Table 5:** Maximum plasma concentration of experimentally validated single agents with activity against malaria

Compound	Maximum plasma concentration, C_Max_ (ng/mL)	Molar mass (g/mol)	Maximal dose (nM)	Refs
FUL	12.6	606.772	20.8	[53]
RAL	1	473.584	2.1	[54]
THI	205.5	370.577	554	[55]
MEG	412	384.516	1071	[56]
TAC	15.8	804.018	19.6	[57]
HYD	32	374.904	85.3	[58]
API	341.6 ± 85.2	623.79	547.6 ± 136.6	[59]
CHR	1.85	1183.25	1.5	[60]

The most synergistic compound combinations were tacrolimus-hydroxyzine, tacrolimus-fulvestrant, ingenol-tacrolimus, fulvestrant-megestrol and raloxifene-thioridazine. All of the synergies were occurring at doses above the maximal safe plasma concentrations, which renders the current study more of a proof-of-principle than a method to select therapeutically relevant compound combinations directly. A clear definition of required pharmacokinetics and pharmacodynamics properties will also help with the design of drug regimens for dosing anti-malarial drug combinations [52]. Hence, the synergistic pairs will require further investigations in in vivo models to fully evaluate the possibility of transferring them into clinic. Moreover, the generated dataset can be used as a training set for further compound combination predictions. Moreover, the approach is interesting to be followed up with larger synergy prediction candidates (with a different test set).

Accuracy of the predictions and identification of novel single agents and combinations suggests that the integration of machine learning methods with gene expression data analysis can lead to a robust platform for predicting novel synergistic compound combinations, in this case applied to malaria.

## Additional files

**Additional file 1: Table S2.** Predicted synergistic compound combinations for malaria. The predicted probability of being synergistic for each compound pair in each strain.

**Additional file 2: Figure S1.** Definition of curve classes. Classes of activity [61] were assigned based on growth inhibition curves where -1.1 shows complete curve as well as high efficacy, -1.2 represents complete curve but partial efficacy, -2.1 symbolizes partial curve but high efficacy, -2.2 represents partial curve and partial efficacy and -2.3 represents partial curve, high efficacy but poor curve fit.

**Additional file 3: Figure S2.** Pathway enrichment for selected predicted compounds. a) The Figure represents the amount of enrichment (Z-score) of each pathway. The Y axis represents the pathway indices of pathways listed in Additional file 4: Table S1.

**Additional file 4: Table S1.** Pathway Z-scores for predicted compounds. The table lists pathways from Biosystems that were significantly enriched at least for one of the predicted compounds. The values are Z-scores calculated for each compound compared to background compounds in the training and test set. The Index ID and Biosystems pathway ID is also provided.

## Abbreviations

ACT: artemisinin-based combination therapy
CHR: chromomycin-a3
FUL: fulvestrant
API: apicidin
ING: ingenol
TAC: tacrolimus
JX4: JX-401
RAL: raloxifene
HYD: hydroxyzine
THI: thioridazine
KIN: KIN001-244
PI8: PI 828
MEG: megestrol
MON: monorden
CHE: chelidonine
NCATS: National Center for Advancing Translational Sciences, National Institutes of Health
GEO: Gene Expression Omnibus
GSEA: Gene Set Enrichment Analysis
QC: quality control
TP: true positives
FP: false positives
FN: false negatives
qHTS: quantitative high-throughput screening
