# Supplementary material for: A systematic and prospectively validated approach for identifying synergistic drug combinations against malaria
Source: Malar J. 2018 Apr 11;17:160. doi: 10.1186/s12936-018-2294-5 (PMC5896032; doi:10.1186/s12936-018-2294-5)
Supplement: Supplementary file 1 — Additional file 1: Table S2. Predicted synergistic compound combinations for malaria. The predicted probability of being synergistic for each compound pair in each strain. [file 12936_2018_2294_MOESM1_ESM.pdf]

Table S2: Predicted synergistic compound combinations for malaria. The predicted probability of being synergistic for each compound pair in **each strain.**

**Compound 1**	**Compound 2**	**3D7**	**DD2**	**HB3**
**ING**	**HYD**	0.869	0.699	0.81
**ING**	**KIN**	0.86	0.703	0.805
**ING**	**JX4**	0.859	0.74	0.826
**JX4**	**MON**	0.859	0.531	0.65
**RAL**	**MON**	0.855	0.545	0.817
**RAL**	**MON**	0.855	0.545	0.817
**MON**	**HYD**	0.844	0.478	0.633
**ING**	**TAC**	0.82	0.674	0.854
**FUL**	**RAL**	0.792	0.739	0.825
**FUL**	**HYD**	0.788	0.747	0.734
**API**	**CHE**	0.765	0.731	0.423
**FUL**	**CHE**	0.756	0.742	0.758
**THI**	**API**	0.702	0.764	0.386
**THI**	**JX4**	0.418	0.753	0.297
**THI**	**PI8**	0.31	0.746	0.447
**CHE**	**ING**	0.843	0.724	0.816
**CHR**	**ING**	0.876	0.672	0.853
