# Supplementary figures and images for: A systematic and prospectively validated approach for identifying synergistic drug combinations against malaria
Source: Malar J. 2018 Apr 11;17:160. doi: 10.1186/s12936-018-2294-5 (PMC5896032; doi:10.1186/s12936-018-2294-5)

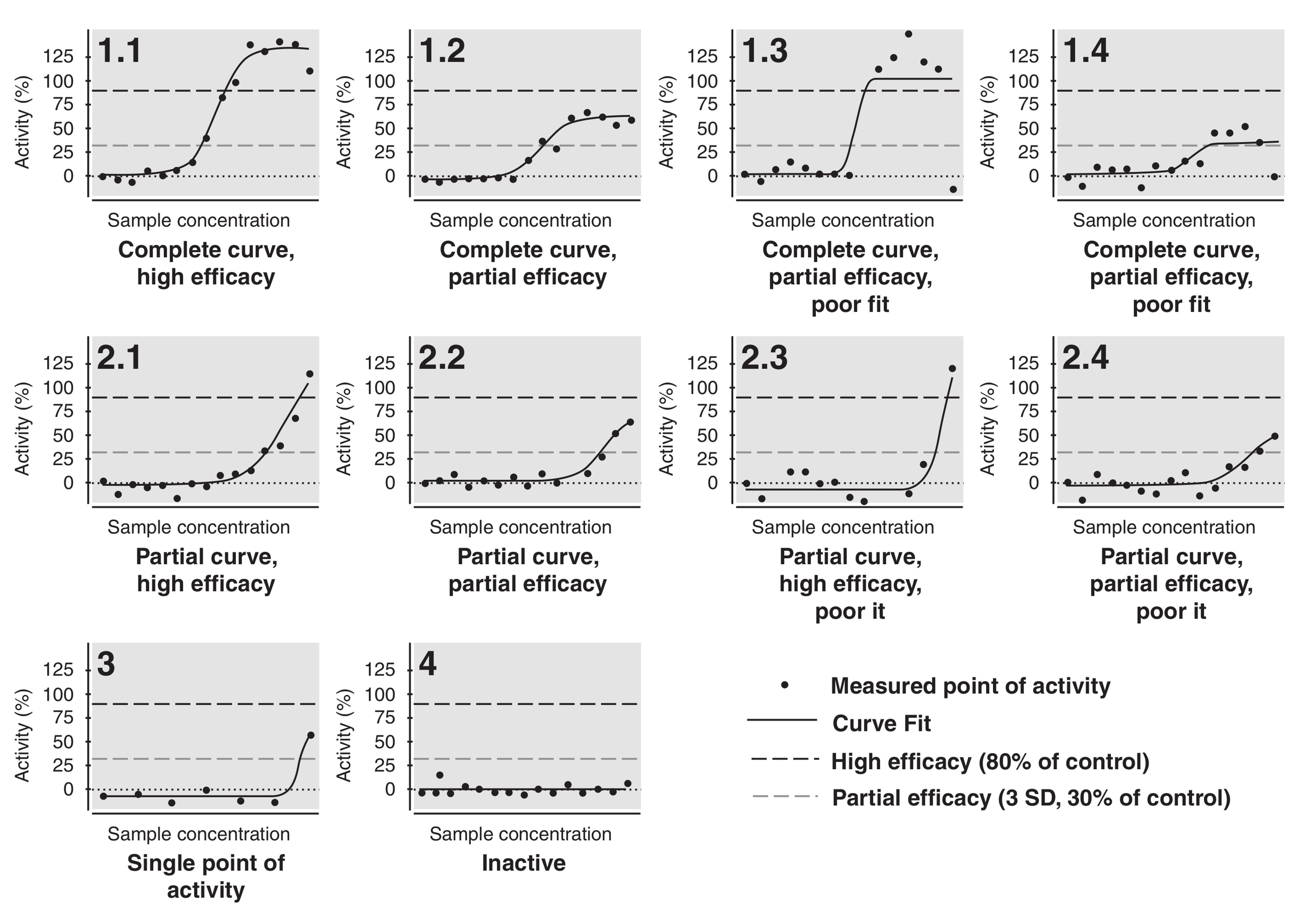

Supplement: Supplementary file 2 — Additional file 2: Figure S1. Definition of curve classes. Classes of activity [61] were assigned based on growth inhibition curves where -1.1 shows complete curve as well as high efficacy, -1.2 represents complete curve but partial efficacy, -2.1 symbolizes partial curve but high efficacy, -2.2 represents partial curve and partial efficacy and -2.3 represents partial curve, high efficacy but poor curve fit. [file 12936_2018_2294_MOESM2_ESM.tiff]

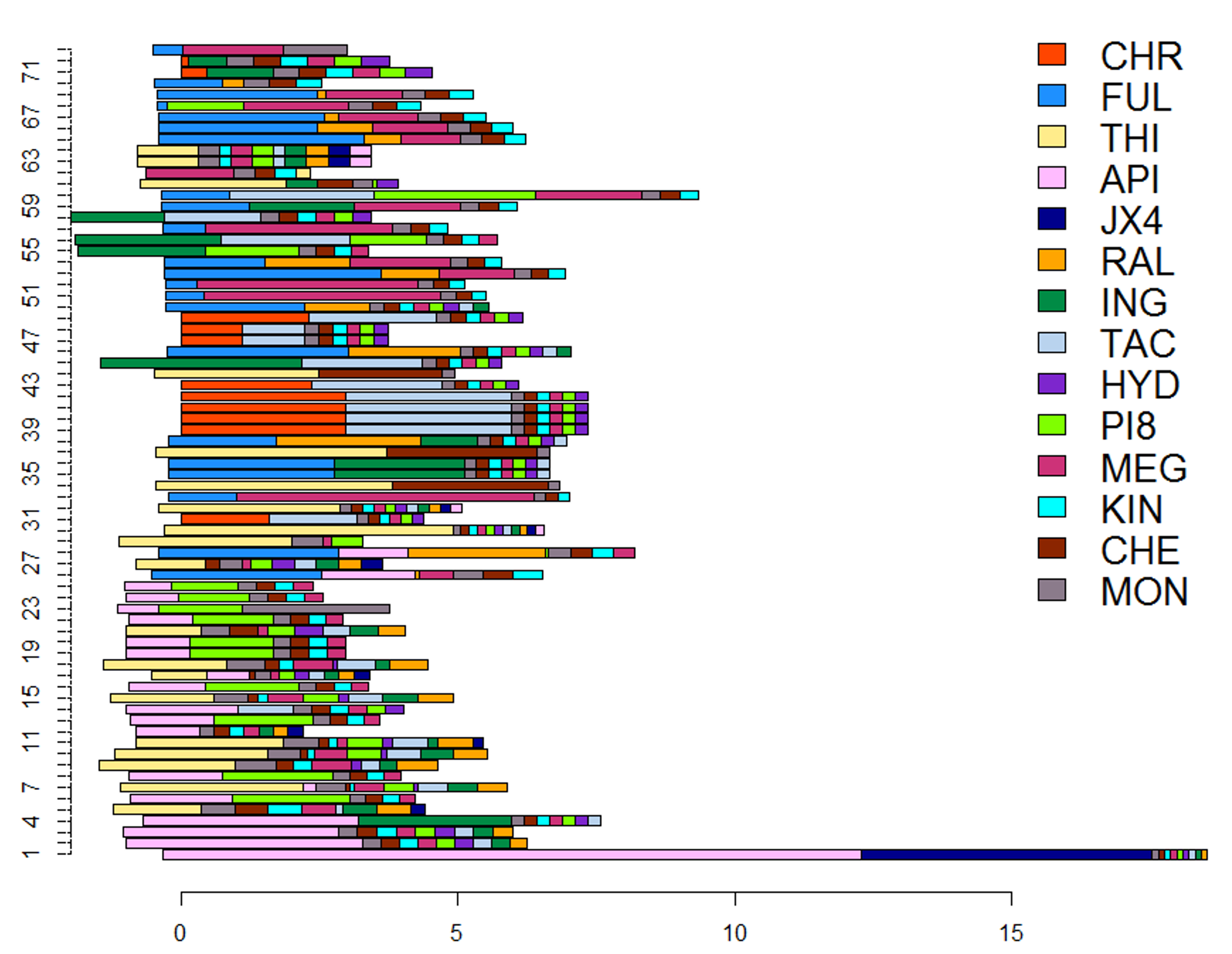

Supplement: Supplementary file 3 — Additional file 3: Figure S2. Pathway enrichment for selected predicted compounds. a) The Figure represents the amount of enrichment (Z-score) of each pathway. The Y axis represents the pathway indices of pathways listed in Additional file 4: Table S1. [file 12936_2018_2294_MOESM3_ESM.tiff]
